# Transcriptional profiling of degraded RNA in cryopreserved and fixed tissue samples obtained at autopsy

**DOI:** 10.1186/1472-6890-6-9

**Published:** 2006-12-04

**Authors:** Andrew C Haller, Deepa Kanakapalli, Rosemarie Walter, Samir Alhasan, James F Eliason, Richard B Everson

**Affiliations:** 1 Wayne State University, Barbara Ann Karmanos Cancer Institute, Detroit, MI, USA; 2 Asterand plc., Detroit, MI, USA; 3 Wayne State University School of Medicine, Departments of Medicine and Pathology, Detroit, MI, USA

## Abstract

**Background:**

Traditional multiplexed gene expression methods require well preserved, intact RNA. Such specimens are difficult to acquire in clinical practice where formalin fixation is the standard procedure for processing tissue. Even when special handling methods are used to obtain frozen tissue, there may be RNA degradation; for example autopsy samples where degradation occurs both pre-mortem and during the interval between death and cryopreservation. Although specimens with partially degraded RNA can be analyzed by qRT-PCR, these analyses can only be done individually or at low levels of multiplexing and are laborious and expensive to run for large numbers of RNA targets.

**Methods:**

We evaluated the ability of the cDNA-mediated Annealing, Selection, extension, and Ligation (DASL) assay to provide highly multiplexed analyses of cryopreserved and formalin fixed, paraffin embedded (FFPE) tissues obtained at autopsy. Each assay provides data on 1536 targets, and can be performed on specimens with RNA fragments as small as 60 bp.

**Results:**

The DASL performed accurately and consistently with cryopreserved RNA obtained at autopsy as well as with RNA extracted from formalin-fixed paraffin embedded tissue that had a cryopreserved mirror image specimen with high quality RNA. In FFPE tissue where the cryopreserved mirror image specimen was of low quality the assay performed reproducibly on some but not all specimens.

**Conclusion:**

The DASL assay provides reproducible results from cryopreserved specimens and many FFPE specimens obtained at autopsy. Gene expression analyses of these specimens may be especially valuable for the study of non-cancer endpoints, where surgical specimens are rarely available.

## Background

Standard microarray analyses use cDNA synthesized from the poly-A tail of RNA. It requires highly intact RNA to be successful. Specimens must be obtained from intact tissues and processed immediately to avoid degradation of the RNA. Avoiding damage is difficult in clinical settings. Autopsy samples may undergo degradation postmortem, surgical samples may undergo degradation during processing, and both may be damaged during storage.

Several recent studies demonstrate that real-time PCR can provide useful gene expression data from clinical specimens, but these assays analyze a single or low number of expression products. It was shown that gene expression analysis obtained with reverse transcription PCR from formalin-fixed, paraffin-embedded (FFPE) tissues is accurate and reproducible, and that results were comparable to those obtained with matching fresh cryopreserved tissue [1]. Also observed was that neither fixation grade nor delay in the formalin fixation of the tissue significantly affected the results. Similar results were also seen by Abrahamsen et al [2]. It was observed that a delay in freezing reduced the amount of total RNA detected, but time in the fixative seemed to have no effect on relative expression of mRNA. While informative, the small number of genes that can be assayed at one time makes assessing gene expression by qRT-PCR laborious and expensive, limiting is usefulness in phases of clinical research and medicine where a broader survey of gene expression is appropriate.

A recently developed cDNA-mediated Annealing, Selection, extension, and Ligation (DASL) assay (Illumina, San Diego, California) promises to meet the needs for highly multiplexed analyses of degraded RNA [3,4]. The DASL assay is based on highly multiplexed PCR amplification rather than hybridization. It requires only short (<50 bp) fragments of RNA, allowing analysis of highly degraded RNA including cryopreserved and FFPE tissues. In the assay 1536 RNA probes allow the selection of a specific set of 512 genes. The assay includes up to three probes per gene to increase its sensitivity in the analysis of informative genes. Between 16 and 384 specimens can be assayed in an experiment using procedures that can be automated and reagent costs much lower than a standard microarray [5].

In addition to the archived FFPE samples, frozen autopsy samples represent a wealth of material as yet underutilized by researchers. Many of these samples sit in freezers and are considered too degraded for standard microarray analyses. With the DASL assay we are able to show replicable results attained from degraded specimens such as these by comparing signal intensities of gene expression targets.

## Methods

### Tissue specimens

Anonymous autopsy tissue specimens obtained following informed consent by next of kin were provided by Asterand plc. (Detroit, Michigan). Autopsies were performed following a standardized protocol so that specimens were collected in less than eight hours of death. Each specimen was cut in half to make two mirror image specimens, one of which was placed immediately in liquid nitrogen and the second was placed immediately in buffered formalin fixative. The fixed tissue was processed using an automated processor (Thermo Shandon, Waltham, Massachusetts) and embedded in paraffin. The blocks were mounted and sectioned using standard histopathologic procedures.

### Extraction, quantification, prequalification

RNA from FFPE tissue specimens was extracted from two 5 *μ*m sections from tissue blocks using the High Pure RNA Paraffin Kit (Roche Diagnostics, Penzberg, Germany) according to the manufacturer's protocol. This procedure included an initial deparaffinization with xylene, followed by an ethanol rinse and drying. The resulting tissue pellet was digested with proteinase K and incubated overnight. The RNA solution was applied to a miniature column, washed, and eluted into a microcentrifuge tube using buffers provided by the manufacturer. The eluant was treated with DNase, followed by a second Proteinase K digestion, and applied to an additional column. The RNA on the column was washed again before being eluted. Typically 28–32 uL of eluant were recovered per extraction.

Total RNA from cryopreserved tissues was extracted following a protocol developed by Asterand combining the TRIzol extraction method (Invitrogen, Carlsbad, California) with further cleanup using the RNeasy mini kit protocol (Qiagen, Valencia, California). The tissue was first homogenized in Trizol, then 0.2 mL chloroform per mL Trizol was added, samples were centrifuged and the aqueous phase collected. Then 0.5 mL isopropanol per mL TRIzol was added and the sample was again centrifuged. After discarding the supernatant, the pellet was resuspended in 75% ethanol, centrifuged and resuspended in RNase free water. The RNA was washed on an RNeasy column multiple times and incubated on the column with DNase I for 15 minutes. RNA was quantified from both methods by the Ribogreen RNA Quantization Assay (Molecular Probes, Eugene, Oregon).

The RNA quality was analyzed using RNA 6000 Nano Assay for the Agilent 2100 Bioanalyzer (Agilent Technologies, Palo Alto, California) by Asterand. All samples were graded "Passed" or "Failed" based on a grading system developed by Asterand. The RNA for the cryopreserved samples was graded from zero to five based on interpretation of the bioanalyzer profiles. The following criteria from the Agilent profiles are used for grading RNA quality. If there is no measurable RNA, the grade is 0. For those samples with measurable RNA, the following criteria are each given 1 point for a total score between 1 and 5:

1. The ratio of 28S to 18S peaks is equal to or greater than 1.3.

2. The area under the 28S and 18S peaks combined is equal to or greater than 30% of the total area.

3. The widths of the 18S and 28S peaks are each less than or equal to 4 seconds.

4. There are no distinct peaks between the 28S and 18S peaks or between the 18S peak and the lower marker peak.

5. The area under the degradation peaks is less than the combined areas of the 28S and 18S peaks.

Cryopreserved samples with Grades 3 and above based on this scoring approach were considered "Passing" or qualifying for most gene array technologies and those below Grade 3 as "Failing". This classification is based on unpublished reports that the likelihood of successful analysis by conventional microarrays. In addition, each FFPE specimen was characterized by the grade of its corresponding cryopreserved mirror sample, and divided into groups according to whether the cryopreserved material "Passed" or "Failed".

RNA quality in the cryopreserved specimens was also measured automatically using the RNA integrity number (RIN) value incorporated into the 2100 Bioanalyzer software. This new attribute provides a tool for standardization of RNA quality control, in a user-independent, automated and reliable procedure [6]. The RIN takes into account decreases of signal intensities for the two ribosomal bands and increases in the presence of shorter fragments between the two peaks and below the 18S band.

The FFPE tissues were not graded by the bioanalyzer except by the quality of the representative cryopreserved mirror samples, due to the fact that the fixation process degraded the RNA to an extent that is poorly characterized by the bioanalyzer profiles.

RNA extractions were pre-qualified for expression analysis by a real-time PCR assay recommended by Illumina Inc. [4]. RNA was reverse-transcribed into cDNA using the Master Mix for cDNA synthesis, single use reagent (Illumina Inc). cDNA was derived from 200 ng of RNA and was amplified with primers 5'-GTACGCTGTGAAGGCATCAA-3' and 5'-GTTGGTGTTCATCCGCTTG-3' to yield a 90 bp transcript-specific fragment of a highly expressed ribosomal protein gene, [GenBank accession # NM_012423.2] *RPL13a*. Amplifications were performed with the recommended PCR program, using a commercial SYBR Green PCR master mix (Applied Biosystems, Foster City, California) and monitored in real-time with a Bio-Rad Icycler and optical head (Bio-Rad Laboratories, Hercules, California).

### DASL gene expression

**Figure 1:**
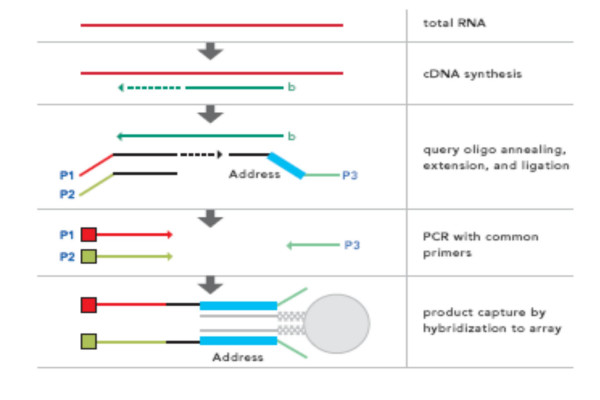
**DASL RNA Profiling Technique **(From [5]).

In the DASL assay (Figure 1) total RNA is converted into cDNA in a reverse transcription reaction using biotinylated oligo-d(T)18 and random hexamers. Pairs of query oligos, up to three unique pairs for each of 512 genes, are annealed to complementary sequences (~50 bases) flanking the specified cDNA target site. The biotinylated cDNA is then bound to streptadadivin particles and mis-hybridized and non-hybridized oligos are washed away. Through a primer extension and ligation process the biotinylated ~100 bp DASL product is formed. This product is then amplified by universal fluorescent primers using conditions detailed in [4] to fluorescently label and amplify the templates generated in the pre-PCR process. The 5' primers (P1 and P2) are labeled with fluorescent Cy3 and Cy5, respectively; while the 3' primer (P3) contains in addition to its primer sequence an address sequence which is complementary to a secondary address sequence located on the Sentrix BeadChip. The double-stranded PCR products are then isolated and hybridized to the BeadChips [3,7].

The BeadChip is composed of 16 individual arrays. The arrays were formed on the slide by densely etching pits designed to hold silica beads. Each array contains about 50,000 3 μm silica bead derivatized with one of 1,536 different sequences. The beads are positioned randomly, and a decoding procedure is used to identify the location and DNA sequence on each bead [8]. After hybridization, the array is then scanned by laser confocal microscopy using an automated BeadStation™ Reader and analyzed by SentrixScan™ software from Illumina. The software creates an intensity data file which is used in statistical analysis of the results.

### Cancer gene panel

The DASL Cancer Panel (Illumina Inc.) includes 1536 unique sequence-specific probes. Genes in the panel were selected by Illumina based on their importance in previous studies. Three probes per gene are used for most genes of interest; allowing for 512 different genes. Probes for each target within the gene were designed by a proprietary algorithm developed by Illumina. DASL assays performed with human genomic DNA as template validated successful analysis of all but 43 of the 1536 probes; the former were excluded from the expression analysis. All 512 genes were thus represented with at least 2 probes per gene, with most represented by three probes.

### Statistical analysis

Gene expression data were normalized and differential expression calculated using algorithms included in BeadStudio Software (Illumina Inc.). Normalization used robust least squares linear fit with signals of probes which had small relative rank change (<0.05) between conditions. Number of detectable genes represents the genes for which the target sequence signal is distinguishable from the negative controls using a statistical procedure implemented in Beadstudio [9]. Scattergrams representing individual relative gene expression were generated using the Beadstudio program from replicate DASL assays and squared Pearson correlation coefficients were obtained.

## Results and discussion

The RNAs from eleven cryopreserved tissues and 10 formalin-fixed, paraffin-embedded tissues were evaluated. The RNA yields tended to be higher for specimens with a "Passing" Asterand quality grade (Table 1). This difference could be attributed to the relative size differences of the RNA fragments seen in the samples because the RNeasy columns used in extraction do not retain fragments of <100 bp. A similar result was seen in the FFPE study, where tissues that were graded "Failed" RNA in their mirrored cryopreserved samples had an average of 81 ng/uL; whereas those with grades of "Passed" had an average of 120 ng/uL (Table 2). RNA extracts from both sets were obtained in ~30 uL elution volumes. FFPE samples were then pre-qualified by RT – PCR using *RPL13a *primers and protocol according to Illumina recommendations. The cycle threshold for intact total RNA controls had an average of ~26 cycles, while the RNAs from clinical specimens amplified in 32 or fewer cycles, thus all specimens passed pre-qualification which requires that the threshold cycles of the test sample be with seven cycles of control sample.

**Figure 2:**
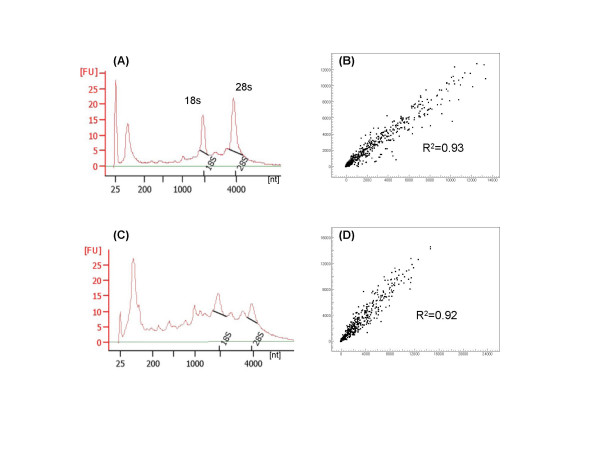
**Comparison of Intact and Degraded Cryopreserved RNAs**. **A**. Electropherogram constructed from Bioanalyzer microchip electrophoresis of RNA extracted from cryopreserved tissue graded "Passed" by Asterand quality assessment method. Prominent bands can be seen as sharp peaks at 18S and 28S indicating highly intact RNA. **C**. Sample that did not pass Asterand quality measures. Electropherogram shows an 18S peak larger than the 28S peak and an increased amount of noise throughout the electropherogram. **B. & D**. The panels on the right show scattergrams of the signal intensities of individual genes for replicate analyses of each sample, and the associated Pearson correlation coefficient squared (R^2^). Both samples provided reproducible results.

Bioanalyzer quality measures and scattergrams of the probe signal intensities for cryopreserved specimens are illustrated in Figures 2. Intact samples have clear 18S and 28S ribosomal RNA peaks with low amounts of noise between them and no other peaks in the area before the 18S peak (Fig. 2A). Degradation of RNA leads to 28S peaks smaller than the 18S peak (Fig. 2B) or even loss of both peaks as well as increased noise throughout the electropherograms (not shown). Five samples were graded as "Passing" according to Asterand methods and six samples were graded as "Failing" (Table 1). Little difference could be seen between scattergrams of the replicate analysis of DASL assays "Passing" RNA and the "Failing" RNA (Figure 2). The squared Pearson correlation coefficients (R^2^) were calculated for each sample. The average R^2 ^for samples with failing RNA was 0.91 and that for samples with passing RNA was 0.92. These results show that the DASL assay can be performed reproducibly even on frozen samples having low quality RNA.

**Figure 3:**
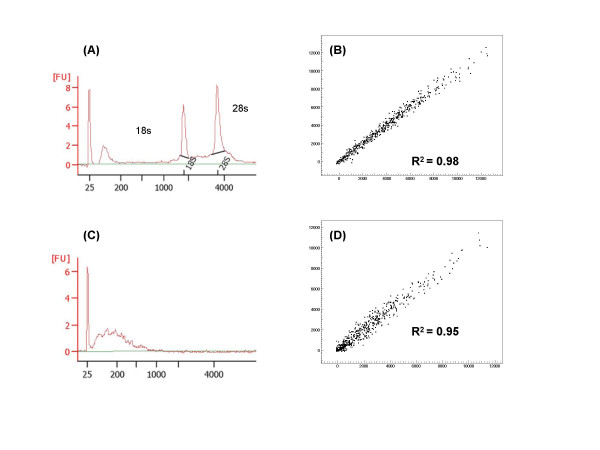
**Comparison of Intact and Degraded FFPE Extracted RNAs**. **A**. Electropherogram of RNA graded "Passed" by Asterand quality measures from cryopreserved tissue of a mirror image preparation of the FFPE sample being studied. One can see clear peaks for both 18S and 28S ribosomal RNA with low amount of noise between the peaks. **C**. Cryopreserved mirror sample graded "Failed" by Asterand quality measure. A lack of distinguishable peaks and also a large amount of fragments in the low molecular weight range indicates a large amount of degradation. **B. & D**. The right shows the reproducibility of the assay based on scattergrams of signal intensities from extracted FFPE RNA on replicate DASL assays and the obtained Pearson R^2 ^correlation coefficient squared (R^2^). Both scattergrams demonstrate highly reproducible results.

Figure 3 presents electropherograms of the cryopreserved RNA sample that is the mirror image of the FFPE samples analyzed, including a sample with highly intact RNA (Passing, Rin = 8.9, Figure 3A) and a sample with degraded RNA (Failing, RIN = 2.3, Figure 3c). Electropherograms from FFPE tissues, regardless of the RNA quality of the mirrored cryopreserved sample, have no distinguishable peaks, with an average fragment length of about 200 nucleotides (not shown). Even with this severe level of degradation before fixation as portrayed in Figure 3b acceptable R^2 ^values can be obtained. Samples with an Asterand grade of "Failing" have a lower average result (R^2 ^= 0.78 < 0.92) versus that of "Passing" RNA (Table 2), but still achieved correlation coefficients as high as 0.95. Overall the R^2 ^values are lower than we have seen in other analyses (data not shown), a possible reason for this may be due to the specimens being refrozen multiple times and samples being run on a number of separate chips.

Cryopreserved specimens from autopsies and FFPE RNA specimens contain a wealth of potential information previously unavailable for highly multiplexed gene expression analyses. Currently, researchers using highly multiplexed gene analyses are limited to working with only the highest quality cryopreserved tissues. Huang et al. showed that with standard array procedures the ischemic time associated with surgical extirpation causes significant differences in gene expression [10]. A similar effect was demonstrated in a study involving post mortem interval and RNA integrity from rat brain tissue. Brain tissue was removed at increasing post mortem time points; subsequently RNA quality was evaluated by 28S/18S ribosomal RNA ratio and gene expression was assessed with cDNA microarray. RNA degradation was seen to increase and amount of mRNAs detected on the microarray decreased with increasing post mortem interval [11].

In contrast, work with qRT-PCR, which requires only small fragments for analysis, suggests much less effect of ischemia or storage. Two studies have looked at RNA degradation in non-fixed human specimens of tonsil, colon, and breast tissue [12,13]. In both studies respective tissues were surgically removed and kept at room temperature for a variety of time points. RNA quality was assessed after extraction by microchip electrophoresis or gel electrophoresis. Gene expression from RNA extracted from these was assessed using RT-PCR analysis. Both studies show stable gene expression across the time points.

Results from the DASL assay, which like qRT-PCR requires only short fragments of RNA, were similar to the qRT-PCR; reproducible results were able to be obtained with cryopreserved specimens over a broad range of sample quality. Bibikova et al has previously shown that reproducibility of the DASL in gene expression profiling compared to that of qRT-PCR. Good correlations were shown between qRT-PCR Cts and DASL signal intensity in fresh- frozen samples (R^2 ^= 0.88) [3]. A lower correlation was observed between qRT-PCR and DASL results for FFPE tissue, which the author attributed to less sensitivity and reproducibility of the qRT-PCR assay.

We further examined whether reproducibility of assay results was influenced by the level of expression of individual genes. Based on signal intensity, the correlation coefficients for replicate arrays was examined for the 100 most highly expressed genes, and compared to the correlation coefficients for the lowest expressed 100 genes for each fixation method. For cryopreserved tissue, the average squared Pearson correlation coefficients were high in both groups, regardless of whether the sample had a passing or failing grade (range 0.64 – 0.82). For the FFPE tissue the correlation coefficients for replicate analyses were acceptable for the 100 most highly expressed genes, but poor for the 100 least highly expressed genes.

The result of this study suggests approaches to validating results obtained from FFPE tissues. Firstly, the reproducibility of the assays could be assessed by comparing results for replicate arrays. Secondly, when assaying FFPE tissue, the replicablity of the array could be increased by excluding lower expressed from the analysis. This would reduce the data obtained from the array, but could be used if running duplicate arrays was not an option. A less expensive pre-qualification measure, and more practical approach to the assaying of these specimens is needed, but the development of such an approach is outside the scope of this study.

## Conclusion

The DASL assay is an innovative microarray based on qRT-PCR technology that allows researchers to conduct high-throughput, highly-multiplexed gene expression array analyses of tissues that otherwise could only be analyzed by qRT-PCR. It provides reproducible results from cryopreserved specimens and many FFPE specimens obtained at autopsy. Gene expression analyses of these specimens may be especially valuable for the study of non-cancer endpoints, where surgical specimens are rarely available.

## Competing interests

RW, SA, and JFE are employees of Asterand plc., whose services include provision of cryopreserved and fixed tissues obtained at surgery and autopsy. The other authors declare no competing interests.

## Authors' contributions

Each author contributed in the research performed to complete this technical report. AH wrote the majority of the article and also performed the data analysis. DK performed the DASL assays. RW was the liaison to Asterand plc and also extracted many of the samples and collected the samples to be run on the Bioanalyzer instrument. SA located the tissues in Asterand's tissue bank and conducted Bioanalyzer analysis. JFE was the supervising investigator at Asterand, and RBE was the principal investigator at Karmanos Cancer Institute. They had overall responsibility for design, development, and conduct of the project. All authors read and approved of the final manuscript.

## Pre-publication history

The pre-publication history for this paper can be accessed here:



## Figures and Tables

**Table 1:** Results for RNA Obtained from Cryopreserved Tissue Specimens

Sample	Type	Asterand Quality Grade	RIN^a^	RNA Conc (ng/μL)	# Genes Detected^b^	Correlation of Replicates (R^2^)^a^
1	Brain	Passed	7.2	550	425	0.88
2	Brain	Passed	8.1	586	411	0.96
3	Brain	Passed	8.1	673	425	0.93
4	Brain	Passed	7.5	635	391	0.91
5	Brain	Passed	6.6	641	391	0.93

	Passing RNA Ave		7.5	617	409	0.92

6	Brain	Failed	3.1	660	395	0.92
7	Brain	Failed	5.8	275	388	0.86
8	Brain	Failed	2.5	740	421	0.92
9	Brain	Failed	6.2	590	415	0.89
10	Brain	Failed	6.5	554	399	0.91
11	Brain	Failed	5.8	598	461	0.93

	Failing RNA Ave		5.0	569	413	0.91

^a^RNA Integrity number as assessed by 2100 Bioanalyzer

^b^Genes detected over background by Beadstudio software with a certainty of p = 0.99

^c^Technical replicates of same RNA extraction run in separate array experiments

**Table 2:** Results for RNA Obtained from Formalin-fixed, Paraffin-embedded Tissue Specimens

			Cryopreserved		FFPE Tissue				
				
Sample	Type	Tissue	Asterand Quality Grade	RIN for Cryo^a^	RIN for FFPE^a^	RNA conc (ng/uL)	PCR Ct^b^	# Genes Detected^c^	Correlation of Replicates (R^2^) ^d^
1	Kidney	Normal	Passed	9.2	2.4	153	30	438	0.91
2	Kidney	Normal	Passed	8.9	2.3	120	25	452	0.98
3	Stomach	Normal	Passed	8.8	2.4	88	31	310	0.86

	Passing RNA Ave			9.0	2.4	120	29	400	0.92

4	Breast	Tumor	Failed	2.3	1.9	119	30	426	0.95
5	Breast	Tumor	Failed	2.5	1.2	71	25	338	0.78
6	Lung	Tumor	Failed	2.4	1.0	54	29	457	0.64
7	Kidney	Normal	Failed	2.4	2.3	87	32	420	0.91
8	Kidney	Normal	Failed	2.7	2.3	61	30	229	0.91
9	Kidney	Normal	Failed	NA	2.6	133	32	377	0.69
10	Kidney	Normal	Failed	2.4	2.4	41	32	164	0.56

	Failing RNA Ave			2.5	2.0	81	30	344.4	0.78

NA = Not Available

^a ^RNA Integrity number as assessed by 2100 Bioanalyzer

^b ^Cycle threshold of amplification of *RPL-13a*, results compared to Ct of intact total RNA

^c ^Genes detected over background by Beadstudio software with a certainty of p = 0.99

^d ^Technical replicates of same RNA extraction run in separate array experiments
